# Supplementary figures and images for: Associative Conditioning Is a Robust Systemic Behavior in Unicellular Organisms: An Interspecies Comparison
Source: Front Microbiol. 2021 Jul 19;12:707086. doi: 10.3389/fmicb.2021.707086 (PMC8327096; doi:10.3389/fmicb.2021.707086)

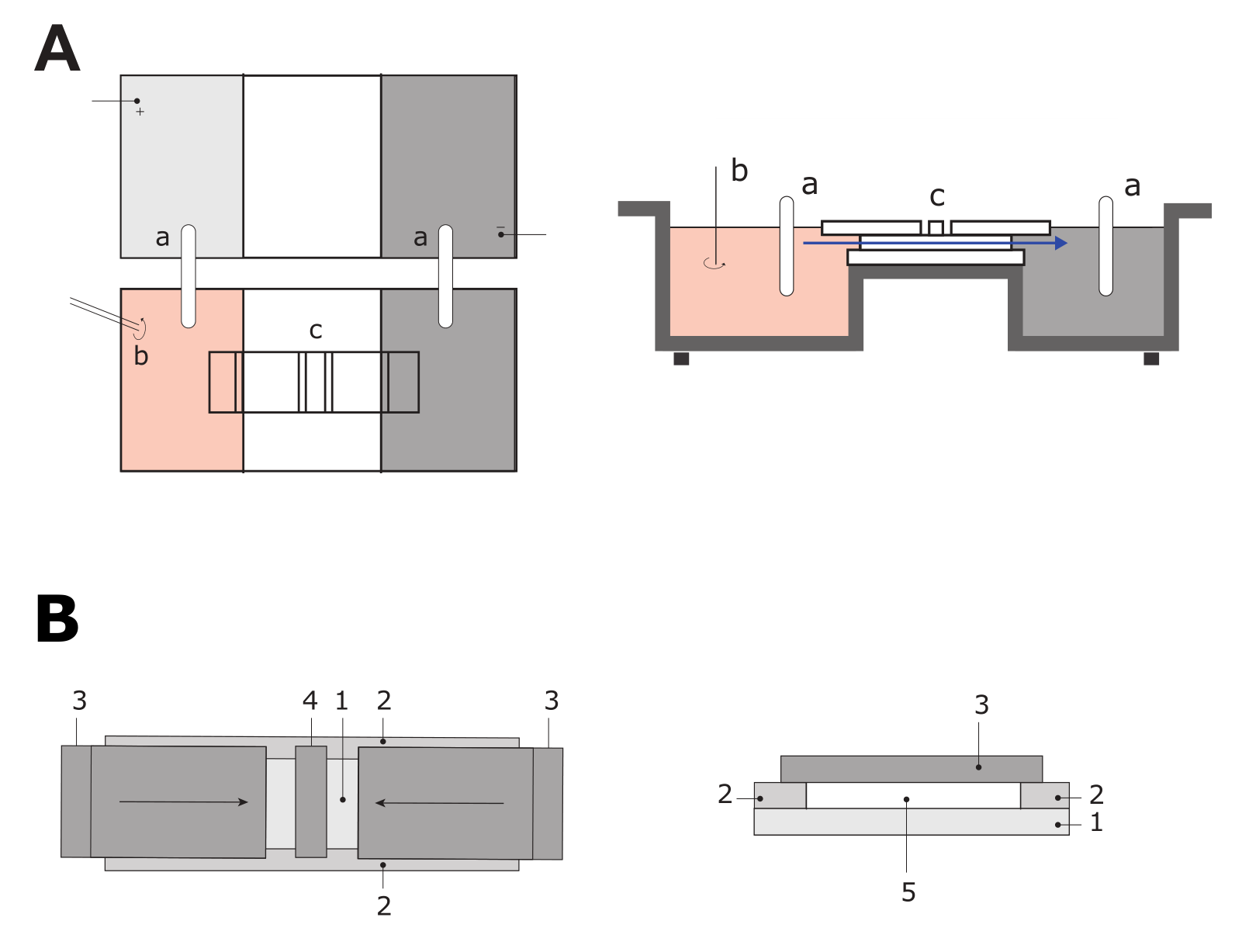

Supplement: Supplementary Figure 1 — Experimental set-up. (A) illustrates the top and lateral views of the experimental system (two standard electrophoresis blocks), in red, the anode where the peptide is initially dissolved, in gray the cathode, a: agar bridges b: mixing pipette c: experimental glass structure; arrow illustrating the flow of the laminar flux. (B) shows the glass chamber where the cells are placed. 1: standard glass slide; 2: longitudinal cover glasses fixed to the glass slide; 3: sliding cover glasses; 4: central cover glass under which the cells are initially placed; 5: experimental chamber where a laminar flux is created and the cells can migrate. [file Image_1.TIF]

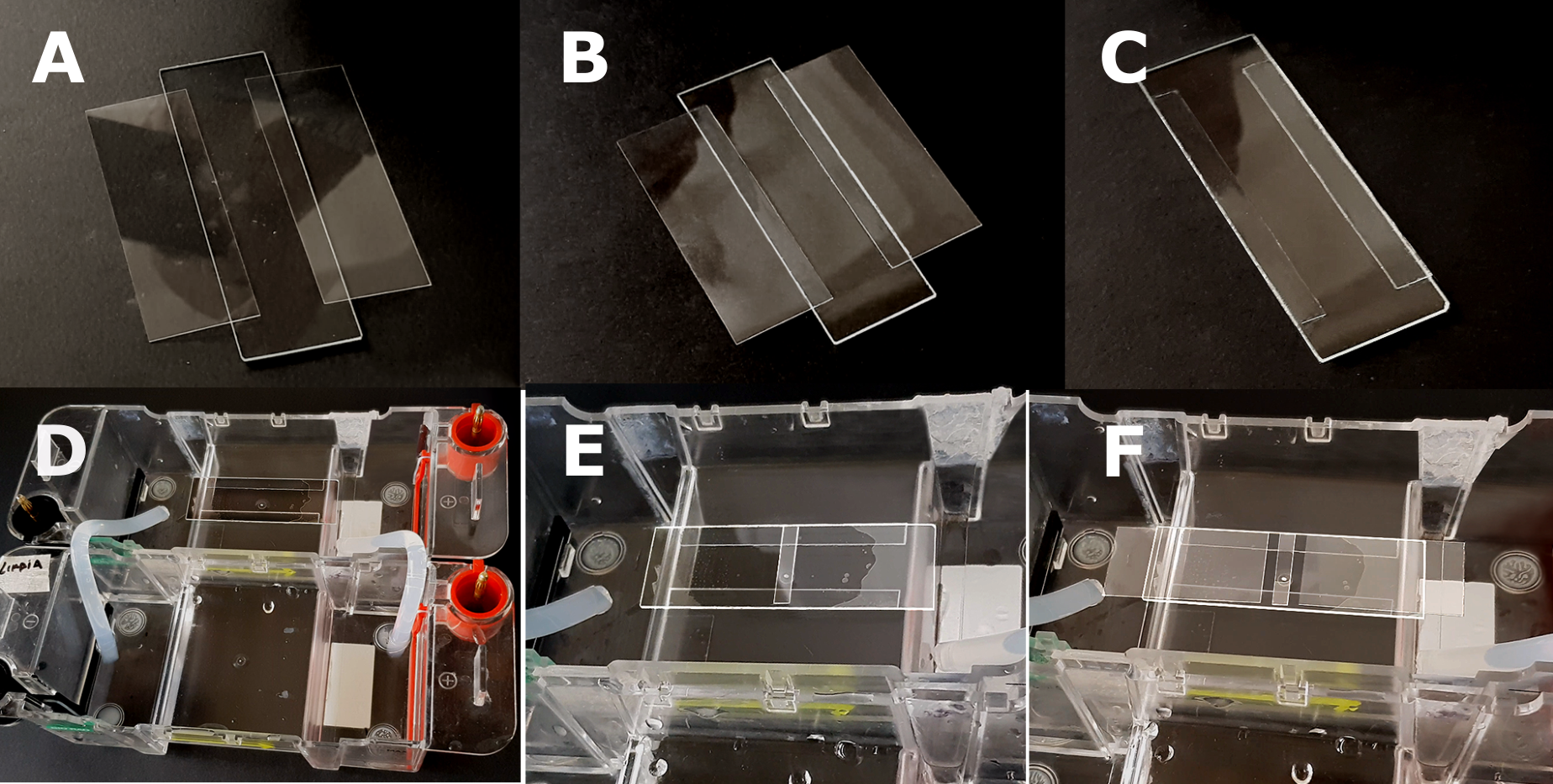

Supplement: Supplementary Figure 2 — Experimental chamber. The specific set-up consists of two standard electrophoresis blocks, two agar bridges and a structure made from a standard glass slide and covers (the experimental chamber) (D–F) The glass structure is composed of one slide and two covers (A). The two covers are fixed to the glass slide with silicone (B), and then they are trimmed with a methacrylate ruler (C). This modified slide is placed in the central platform of the second electrophoresis block (D). To avoid that the medium goes across the modified slide, we placed an oil drop under, it in the central platform of the block of electrophoresis. Finally, a central piece of cover glass about 3 × 24 × 0.1 mm (E) and two sliding lateral cover pieces are placed on the modified slide (F). When the sliding cover pieces are moved closing the central part (see Supplementary Figure 1) an inner laminar flux is generated in the chamber and when they are open, cell placement and rescue are possible under the central piece of coverslips. [file Image_2.TIF]

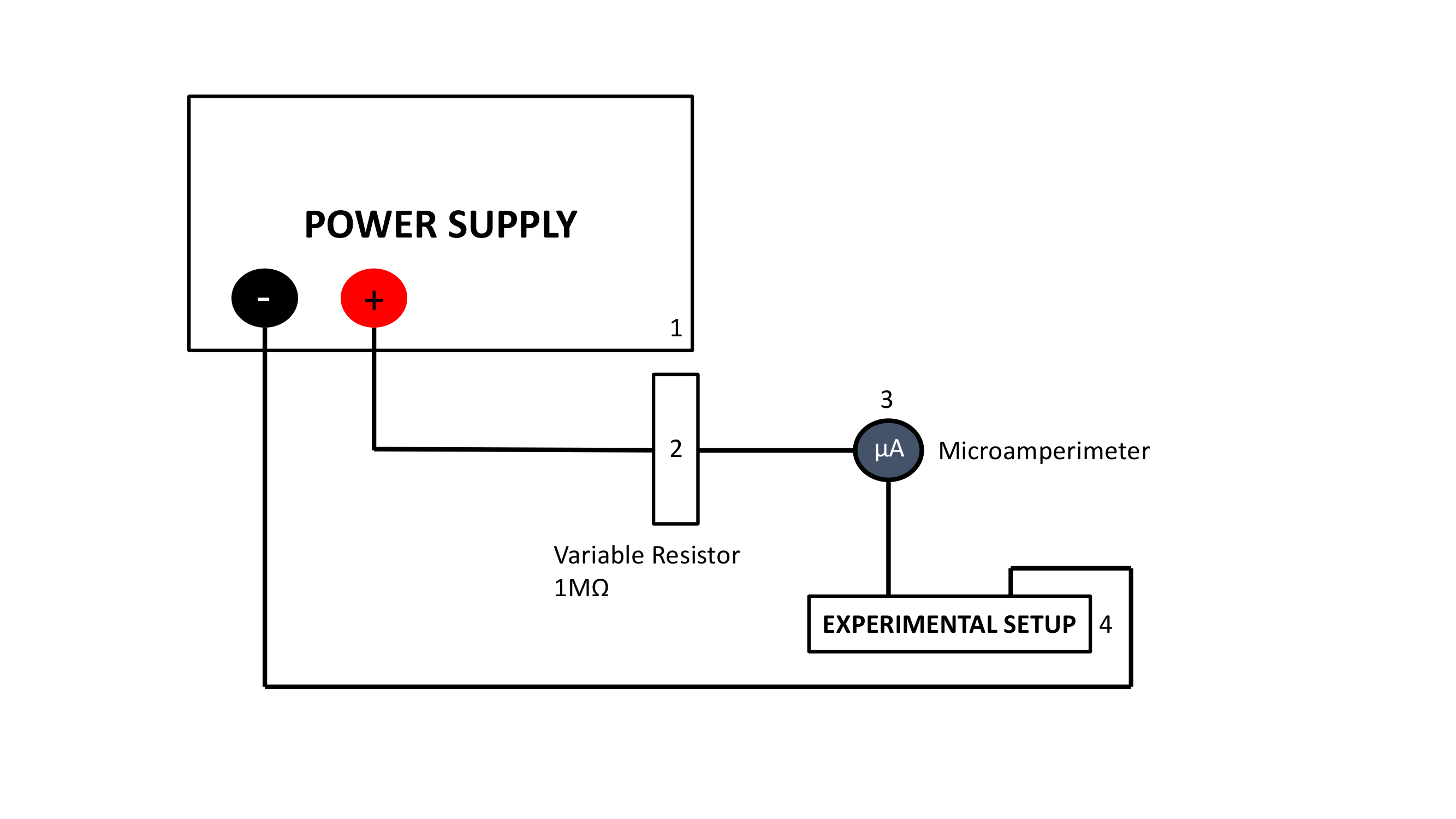

Supplement: Supplementary Figure 3 — Supplementary electric device. The goal of these devices is to regulate and measure the electrical intensity that circulates through the system. 1, Standard Electrophoresis power supply, set at constant voltage. 2, 1 MΩ linear variable resistor that regulates the current of the system. 3, a micro ampere meter connected in series to the system, to measure the current intensity. 4, experimental setup described in Supplementary Figures 1, 2. [file Image_3.TIF]

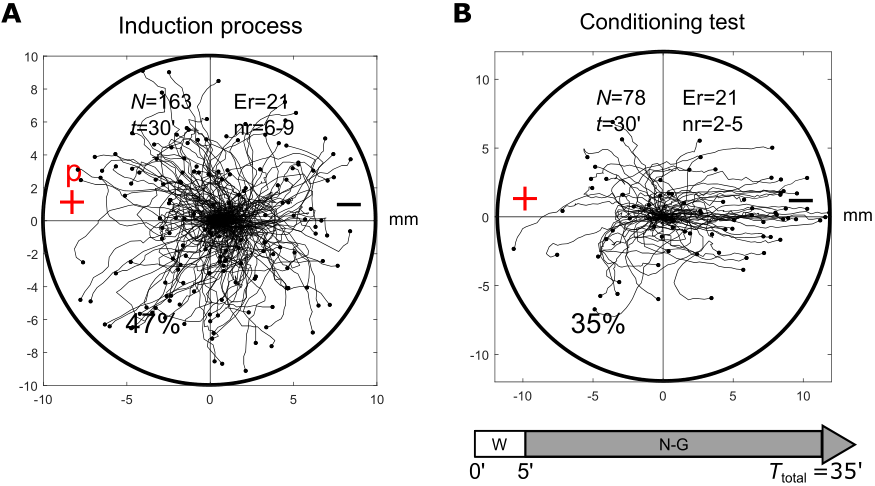

Supplement: Supplementary Figure 4 — Conditioning process in Amoeba proteus using non-optimal electric field intensity. (A) Under simultaneous galvanotaxis and chemotaxis (induction process), 47% of the cells moved toward the anode-peptide (induced cells). These results agree with those obtained when using optimal intensity values for Amoeba proteus (70-74 μA, see Figure 2). (B) After the induction process, the cells were placed in Chalkley’s medium without any stimulus for 5 min, and then they were exposed to galvanotaxis for 30 min using non-optimal electric field intensity values (83-90 μA). 35% of the induced cells presented lasting directionality toward the anode (where the chemotactic peptide was absent) compared to 75% when using optimal intensity values for Amoeba proteus (see Figure 2). However, despite using non-optimal calibration, the cosine values obtained from the conditioned test were significantly different to those from the galvanotaxis (p−value = 10−5), indicating the emergence of a new behavior in the migration patterns. “N” total number of cells, “Er” experimental replicates, “nr” number of cells per replicate, “t” time of galvanotaxis or chemotaxis, “+” anode, “−” cathode. Both the x and y-axis show the distance in mm, and the initial location of each cell has been placed at the center of the diagram. [file Image_4.TIF]

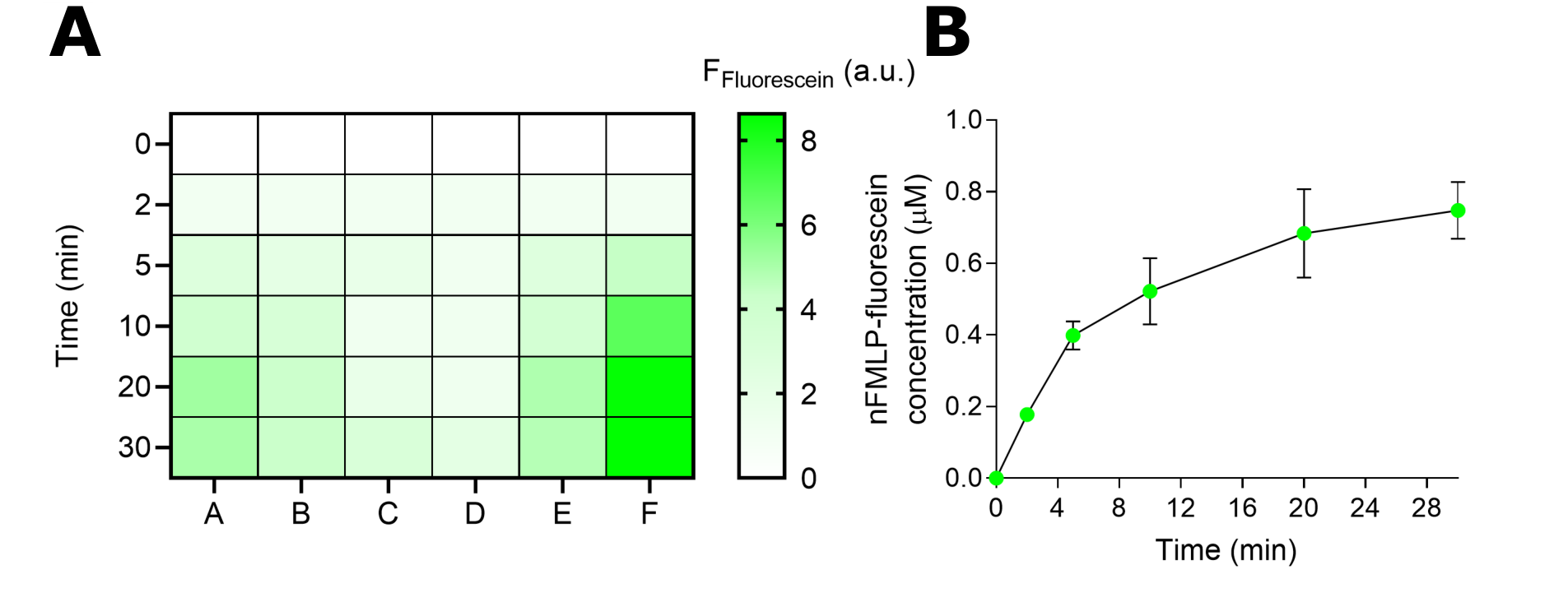

Supplement: Supplementary Figure 5 — Fluorescein-tagged peptide concentration in the middle part of the laminar chamber flux. Fluorescein-tagged peptide concentration in the middle part of the laminar chamber flux as determined with a microplate reader. (A) heat map presenting individual fluorescence measurements at different times. (B) average levels of nFMLP-fluorescein concentration as time-function. The data represent the Mean ± SEM of 6 measurements (A–F, taken at 0, 2, 5, 10, 20, and 30 min). The peptide concentration in the middle of the glass experimental chamber (where the amoebae are placed) increases immediately following the flow establishment (within 2 min the concentration rises from zero to approximately 0.2 μM) and this concentration increases further (to 0.6 μM) for at least 30 min. [file Image_5.TIF]
